# Multisite λ-Dynamics for Protein–DNA Binding Affinity Prediction

**DOI:** 10.1021/acs.jctc.4c01408

**Published:** 2025-03-24

**Authors:** Carmen Al Masri, Jonah Z. Vilseck, Jin Yu, Ryan L. Hayes

**Affiliations:** † Department of Physics and Astronomy, Uninversity of California, Irvine, California 92697, United States; ‡ Department of Biochemistry and Molecular Biology, Center for Computational Biology and Bioinformatics, Indiana University School of Medicine, Indianapolis, Indiana 46202, United States; § Department of Physics and Astronomy, Department of Chemistry, University of California, Irvine, California 92697, United States; ∥ Department of Chemical and Biomolecular Engineering, Department of Pharmaceutical Sciences, University of California, Irvine, California 92697, United States

## Abstract

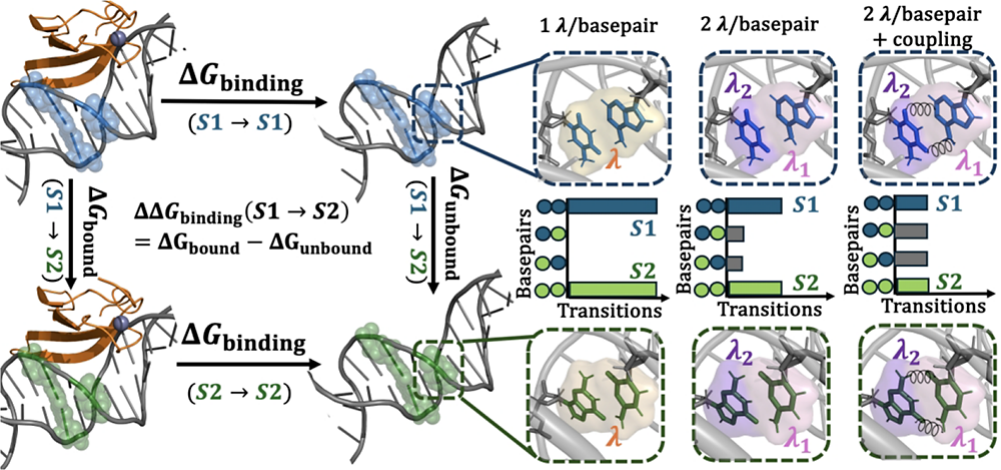

Transcription factors (TFs) regulate gene expression by binding to specific DNA sequences, playing critical roles in cellular processes and disease pathways. Computational methods, particularly λ-Dynamics, offer a promising approach for predicting TF relative binding affinities. This study evaluates the effectiveness of different λ-Dynamics perturbation schemes in determining binding free energy changes (ΔΔ*G*_*b*_) of the WRKY transcription factor upon mutating its W-box binding site (GGTCAA) to a nonspecific sequence (GATAAA). Among the schemes tested, the single λ per base pair protocol demonstrated the fastest convergence and highest precision. Extending this protocol to additional mutants (GGTCCG and GGACAA) yielded ΔΔ*G*_*b*_ values that successfully ranked binding affinities, showcasing its strong potential for high-throughput screening of DNA binding sites.

## Introduction

Transcription Factors (TFs) are DNA-binding proteins that regulate gene expression by selectively binding to specific DNA sequences.^1−4^ These proteins play an essential role in cellular regulatory processes, including both the activation and suppression of genes.^1,2^ Central to many cellular signaling pathways, TFs are implicated in a wide array of diseases, including metabolic disorders^5^ and various forms of cancer.^6−8^ Consequently, identifying TF binding sites and understanding how DNA mutations at these sites impact cellular pathways are crucial for both understanding and treating such conditions.

While experimental techniques like high-throughput SELEX,^9^ protein binding microarrays (PBM),^10,11^ and chromatin immunoprecipitation followed by sequencing (ChIP-Seq)^12,13^ have significantly expanded our knowledge of TF binding sites and binding specificity, the high costs and labor-intensive nature of these methods call for alternate techniques to evaluate DNA binding such as efficient computational strategies for accurately predicting binding affinities. These methods span from machine learning models using DNA sequences and their structural or physiochemical properties^14−20^ to molecular dynamics (MD) simulations utilizing free energy and enhanced sampling techniques.^21−32^

Among these methods, alchemical approaches such as free energy perturbation (FEP),^33−36^ thermodynamic integration (TI),^37^ and λ-Dynamics^38−40^ have emerged as key methods for obtaining thermodynamic measurements from MD simulations. These methods use a variable called λ that governs the progression through chemical space and modulates the potential energy landscape between two distinct states. Unlike FEP and TI, which conduct a series of simulations at closely spaced fixed λ values, λ-Dynamics uses λ as a continually varying parameter within a single simulation, analogous to atomic positions.^38,40^ Consequently, all intermediate states are explored within a single simulation.

Multisite λ-Dynamics, a more recent version of λ-Dynamics,^41^ enables the simulation of multiple mutations at distinct protein or ligand sites simultaneously, significantly reducing the computational expense typically required for such analyses. The method has been enhanced by the adaptive landscape flattening (ALF) method to facilitate faster convergence and soft-core potentials for a higher accuracy of the free energy measurements.^42^ Additionally, the Basic λ-Dynamics Engine (BLaDE)^43^ has allowed for significant improvements of simulation speed on GPUs.

Consequently, Multisite λ-Dynamics has proven to be very efficient, requiring one to 2 orders of magnitude less computational resources than other free energy calculation methods at the same level of accuracy.^41,44^ It is also highly scalable, capable of comparing hundreds of physical systems in a single simulation,^39,45^ and it is capable of accurately predicting the effects of mutations on system stability.^46,47^ Collectively, the speed, scalability, efficiency, and precision of multisite λ-Dynamics make it exceptionally well suited for investigating TF-DNA binding and rapid screening of multiple DNA sequences.

In this study, we evaluated the efficacy of λ-Dynamics in measuring changes in protein–DNA binding affinities. We focused on the WRKY transcription factor domain and its binding affinity changes upon mutation of its specific W-box DNA binding site (GGTCAA to GATAAA). We assessed three different sampling protocols, each characterized by a distinct strategy for varying λ: driving each base-pair transformation by λ, assigning an individual λ to each base, or decoupling the λ variables with additional biases.

Among these, we found that the single λ per base-pair transformation protocol offered the fastest convergence. Following this, we expanded our analysis to mutations of the W-box to two additional nonspecific sequences (GGTCCG and GGACAA). We then compared the resulting ΔΔ*G*_*b*_ values to experimentally obtained binding affinities and use this comparison as a benchmark to validate the predictive power and accuracy of our computational approach.

## Methods

### Multisite λ-Dynamics

**Figure 1:**
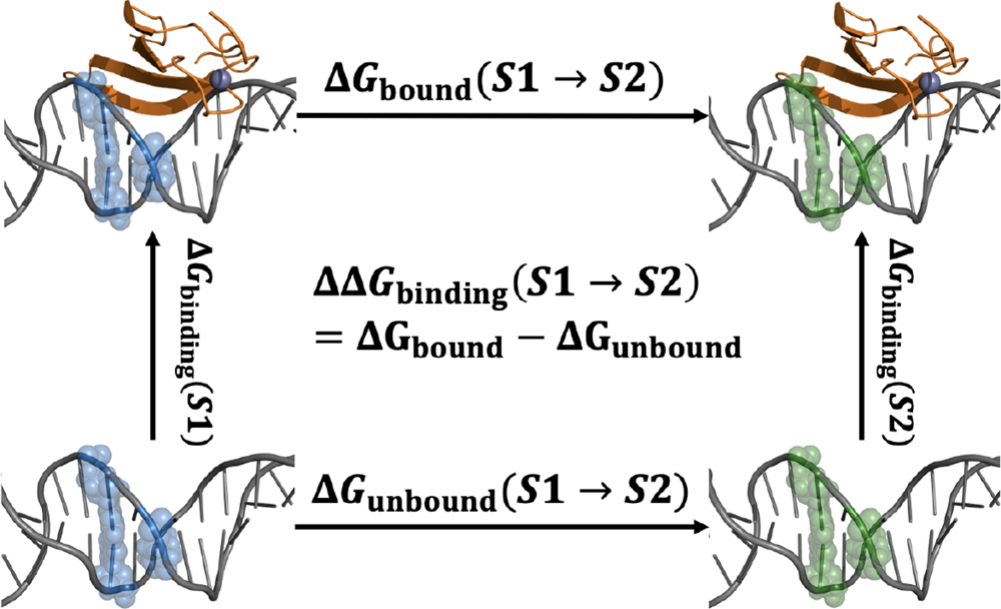
Thermodynamic cycle to estimate ΔΔ*G*_binding_(*S*1 → *S*2), the change in binding free energy upon mutation from sequence *S*1 (blue spheres) to sequence *S*2 (green spheres).

Multisite λ-Dynamics follows a thermodynamic scheme to calculate free energy differences, as shown in Figure 1. Given that free energy is a state function, the binding free energy change when DNA sequence *S*1 is mutated to *S*2 can be estimated in two ways:
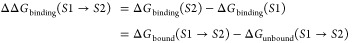
1

The first expression converges slowly due to the multitude of binding pathways that are difficult to sample during typical simulation time scales. While enhanced sampling methods such as Steered MD,^48,49^ Umbrella Sampling,^50^ Metadynamics,^51^ and Gaussian Accelerated MD^52^ can accelerate convergence, they do not eliminate the issue entirely. Additionally, finding the appropriate reaction coordinate or collective variable remains a significant challenge.^53−56^ In contrast, the second expression converges rapidly and can be readily obtained by alchemically transforming *S*1 to *S*2 in both the bound and unbound states. This approach leverages the efficiency of alchemical transformations to provide accurate and computationally feasible estimates of binding free energy changes.

The alchemical transitions between *S*1 and *S*2 are achieved by varying the coupling parameter λ, which tunes the potential energy function from sequence *S*1 at λ = 0 to sequence *S*2 at λ = 1 through nonphysical intermediate states. Multisite λ-Dynamics facilitates the analysis of several mutations across various locations by employing a composite potential function defined as
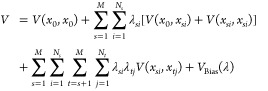
2Here, *s* and *t* denote the DNA site indices that will be mutated, and *i* and *j* represent one of the substituent indices. *x*_0_ denotes the atom positions common to all configurations, including solvent, whereas *x*_*si*_ specifies the particle positions at site *s* for substituent *i*. *M* and *N*_*s*_ stand for the total number of sites and the number of substituent at site *s*, respectively. *V*_Bias_(λ) is a bias potential applied to the λ coordinates to improve sampling efficiency. *V* (*x*, *y*) describes the interaction potential between two atom groups *x* and *y*. The terms , , and  represent the self-interactions of the environment, the interactions of substituent *i* at site *s* with the environment, the self-interactions of substituent *i* at site *s*, and the interaction potential between substituents at different sites, respectively. To preserve the geometry and connectivity of mutations at the noninteracting λ = 0 state, the bonds, angles, and improper dihedrals are not scaled by λ.

Given that fluctuations in alchemical λ space only become statistically independent following successful barrier crossings between λ = 0 and λ = 1, accurately determining the relative free energy between two states critically depends on achieving numerous transitions between these end points. The biasing potentials *V*_*Bias*_ are used to lower the energy barriers in the transition region, facilitating many transitions between end points within a single simulation. The form of these potentials is the same as in previous works^42,46^ and is summarized in Supplementary Text S–I. The ALF^42^ algorithm is used to tune these potentials to obtain a flattened energy landscape, improving the efficiency of sampling increasing transitions between endstates and increasing the precision of the free energy predictions. More details on the ALF protocol are provided in the “Flattening the Free Energy Landscape using ALF” section.

To ensure that only one substituent at each site interacts with the rest of the system at each of the physically relevant end points (i.e., λ_*si*_ = 1 for one substituent *i* at each site *s*, and all remaining λ_*sj*_ = 0 for *j* ≠ *i*), we use the following constraints:
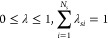
3These constraints are maintained using implicit constraints, as in previous works.^41,42^ The binding free energy difference between two protein–DNA systems can then be approximated as
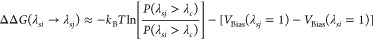
4where  is the probability within the simulation of finding  for all *M* substituents associated with a particular ligand. As λ_*c*_ approaches 1, this equation becomes exact. Here, λ_*c*_ = 0.99 was used to bin the states.

### DNA Perturbation Strategies

As mutating a DNA site requires mutating the corresponding base pairing site on the opposite strand, three strategies of varying the coupling parameter λ were evaluated:

(1) Each base pair transition is scaled by a single λ (i.e., 1 λ for each base pair was used).
(2) Each base transition is scaled by one λ (i.e., 2 λ variables for each base pair).
(3) Each base transition is scaled by one λ, but an extra bias coupling the two λ variables is tuned to decouple transitions.

In the third approach, we introduced a coupling bias to decouple the λ variables across different sites. This coupling bias is applied through a quadratic potential *V*_Quad_ (see Supplementary Text S–I) as follows:
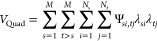
5where Ψ_*s*1,*tj*_ = 0 and Ψ_*si*,*t*1_ = 0. This specific form of *V*_Quad_ represents the interaction between substituents on separate sites *s* and *t*. We implemented this coupling because the high energy associated with mutating one base but not its complementary base creates a barrier to the transitions of the base pair, thus coupling the λ values should enhance transition rates, which facilitates convergence.

### Simulation Details

Our starting structure was WRKY1-N in complex with a W-box DNA, taken from the crystal structure (PDB: 6J4E). Following the protocol by Dai et al.,^54,57^ we extended the 15-bp DNA construct to 34 bp to avoid DNA end-effects in the simulations. The CYS and HIS residues in the WRKY protein were patched to CYM (deprotonated state) and HSE (residue 164, with ϵ-nitrogen protonated) or HSD (residues 138 and 166, with γ-nitrogen protonated), respectively, to form a stable zinc finger domain in CHARMM. The system was solvated in a cubic box with a minimum distance of 12 Å on each side using TIP3P water.

Simulations were conducted using the CHARMM molecular simulation package,^58,59^ developmental version 49a1, utilizing the BLaDE^43^ engine within CHARMM. The CHARMM36m^60^ protein force field and CHARMM36^61^ nucleic acid force field were employed for all simulations. A time step of 2 fs was used, with hydrogen bond lengths constrained via the SHAKE^62^ algorithm. van der Waals interactions were switched off between 9 and 10 Å, and Ewald electrostatics^63^ with κ = 0.320 were applied for long-range interactions.

Prior to production runs, steepest descent minimization was performed for 200 steps with heavy DNA and protein atoms fixed, followed by another 200 steps with harmonic restraints (force constant 10 kcal/mol/Å^2^). The system was then equilibrated for 10 ns using the Langevin thermostat and Monte Carlo barostat, maintaining a temperature of 298 K and a pressure of 1 bar. The mutations studied included the W-box sequence GGTCAA mutated to GATAAA, GGTCCG, and GGACAA. To implement these mutations, a hybrid topology was employed, where the ribose and phosphate backbone were common to both states, and multiple overlapping nucleobase substituents were present. The partial charges of the phosphate and ribose at the end states were identical and always summed to zero, eliminating the need for any additional corrections. A figure showing the hybrid construct is shown in Figure S1.

### Flattening the Free Energy Landscape Using ALF

ALF was employed to flatten the free energy landscape. A general overview of the protocol is provided in Supplementary Text S–II. The procedure to optimize the parameters within *V*_Bias_ was applied as follows. Initially, all biasing parameters in *V*_Bias_ were set to zero, and iterative flattening simulations of up to 100 ps were conducted. After each simulation, parameter estimates were updated based on the free energy profile obtained using WHAM,^64^ with uncertainties calculated as the standard deviation from bootstrapping. Subsequently, up to ten iterative 1 ns simulations were performed to further refine the parameters using the same methodology. Finally, four production runs of varying duration (5, 20, 20, and 100 ns) were executed using the optimized biasing potentials, with further refinement after each run. However, the GGTCCG mutant required additional flattening. Free energy profiles indicated that moderate barriers remained in the alchemical energy landscape, which required further flattening before proceeding to longer simulations. For the unbound DNA, the flattening procedure remained the same, but six production runs of 5 ns duration were conducted, followed by one production run lasting 20 ns and a final run of 100 ns. For the bound DNA, an extra 15 rounds of 1 ns flattening were required, followed by three production runs of 5 ns each, one of 20 ns, and a final run of 100 ns.

## Results and Discussion

### Choosing the Optimal Perturbation Scheme

Each of the three perturbation schemes detailed in the Methods section has its own advantages and limitations. The first scheme (1λ) involves using a single λ to govern the mutation of each selected base pair. This method prevents base mispairing and focuses on transitions between states of interest. However, it involves relatively large perturbed groups (approximately 20 heavy atoms), which may introduce larger energy barriers that can impede transitions between end states, potentially reducing sampling efficiency.

In contrast, the second scheme (2λ) applies a single λ to each individual base. This approach allows for the exploration of a broader conformational space, potentially facilitating faster convergence. However, because the bases in the DNA are paired, the high energy of mutating one base but not the other could create a barrier to transitions forming the base pair, making convergence harder. Additionally, this method risks extensive sampling of undesirable states, including those with base mismatches and structural distortions, leading to inefficient use of computational resources. To address the transition limitations of the second scheme, the third perturbation scheme (coupled 2λ) introduces intersite coupling bias between λ values while still scaling each base by a separate λ. This scheme aims to improve sampling efficiency and foster effective transitions to the final mutated sequences. However, it also increases the risk of sampling noncanonical base pairs as intermediary states between base pairs because the coupling term favors them to enhance transition rates.

**Figure 2:**
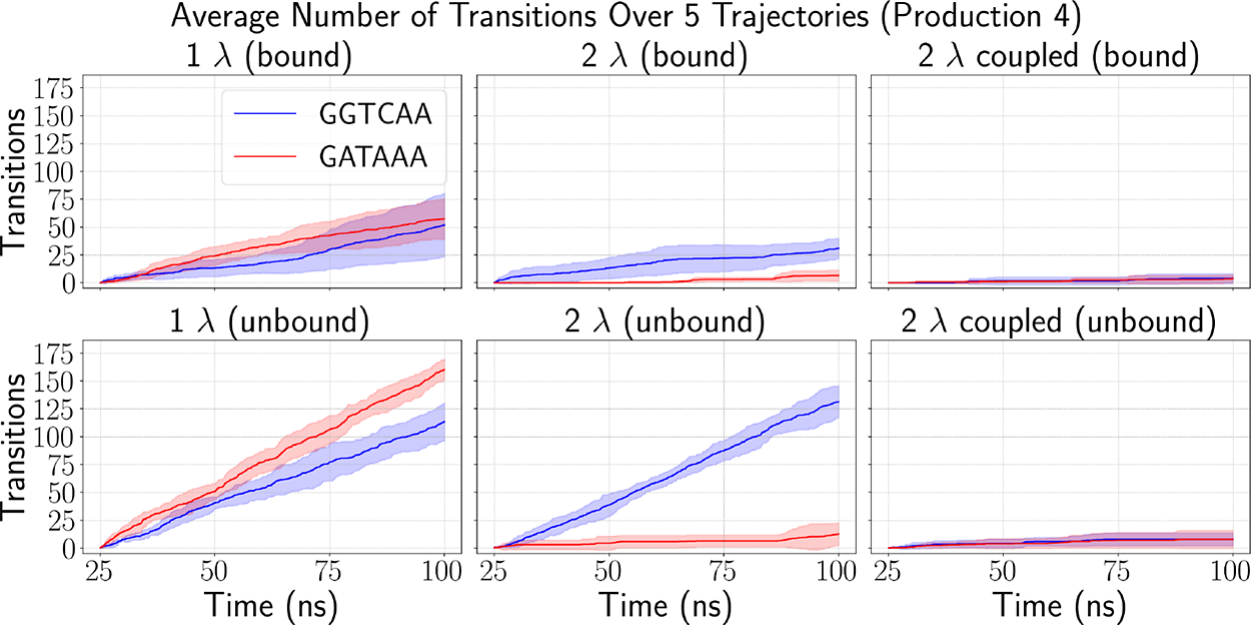
Average number of transitions to the specific DNA sequence GGTCAA (blue) and the nonspecific GATAAA sequence (red) is shown for all three perturbation schemes during the final 100 ns production run. The shaded region represents the standard deviation across the 5 trajectories from each run. Results for the bound system are shown in the top row, while those for the unbound DNA are shown in the bottom row.

The number of transitions between end states achieved within a single simulation can serve as a reliable estimate of the convergence of free energies and, consequently, the effectiveness of a perturbation scheme. To evaluate the perturbation schemes, we analyzed transitions from the specific W-box sequence (GGTCAA) to a nonspecific sequence (GATAAA) across four consecutive production runs (labeled as Production 1–4). Each run comprised five trajectories. The results for Productions 1–3 are detailed in Supplementary Figure S2a–c, and those for Production 4 are depicted in Figure 2. We found that even from the first production run (Figure S2a), the 1λ scheme showed an advantage over the other two schemes by allowing multiple transitions to both GGTCAA and GATAAA. In contrast, the other schemes failed to achieve transitions to at least one of the states. Production 2 (Figure S2b) showed a similar trend, with improved sampling of the unbound GATAAA in both the 2λ and the coupled 2λ schemes.

By Production 3 (Figure S2c), all schemes exhibited transitions to every state, but the number of transitions was the lowest in the coupled 2λ scheme. While the 2λ scheme had comparable transitions for the mutant GATAAA to the 1λ scheme, transitions to the native GGTCAA were significantly lagging. Finally, in Production 4 (Figure 2), the 1λ scheme maintained a consistent transition rate of approximately 0.5 transitions/ns in the bound system and 1 transition/ns in the unbound system for each target state. These rates were consistent from Production 1, indicating rapid bias convergence. In contrast, transition frequencies in the other two schemes varied across production runs, implying biases that have not yet converged well.

Additionally, both in the uncoupled and coupled 2λ schemes, most of the states had limited transitions. This was particularly true for the coupled 2λ scheme, where the total number of transitions remained below 10. This seemingly poor performance can be explained by looking at how transitions are distributed across physical states in Supplementary Figure S3: While the total number of transitions was comparable to the uncoupled 2λ in the unbound state and to the 1λ in the bound state, transitions were evenly spread across all states in the coupled scheme, resulting in undersampling of the states of interest.

Conversely, in the uncoupled 2λ scheme, the native W-box and GATCAA states were favored while the rest of the states had little to no transitions. This limited sampling suggests that, despite flattening the free energy profile, the biases are not sufficiently converged and the system remains biased toward more favorable states. This bias most likely stems from the intrinsic coupling between residues, which the scheme lacks the appropriate bias to address. As a result, the system struggles to effectively transition to other physical states. Additionally, even though the free energy is equalized, slower diffusion in certain parts of the chemical space may lead to reduced transition rates, preserving the overall probability distribution but hindering adequate sampling of all states.

### Free Energy and Uncertainty Convergence

**Figure 3:**
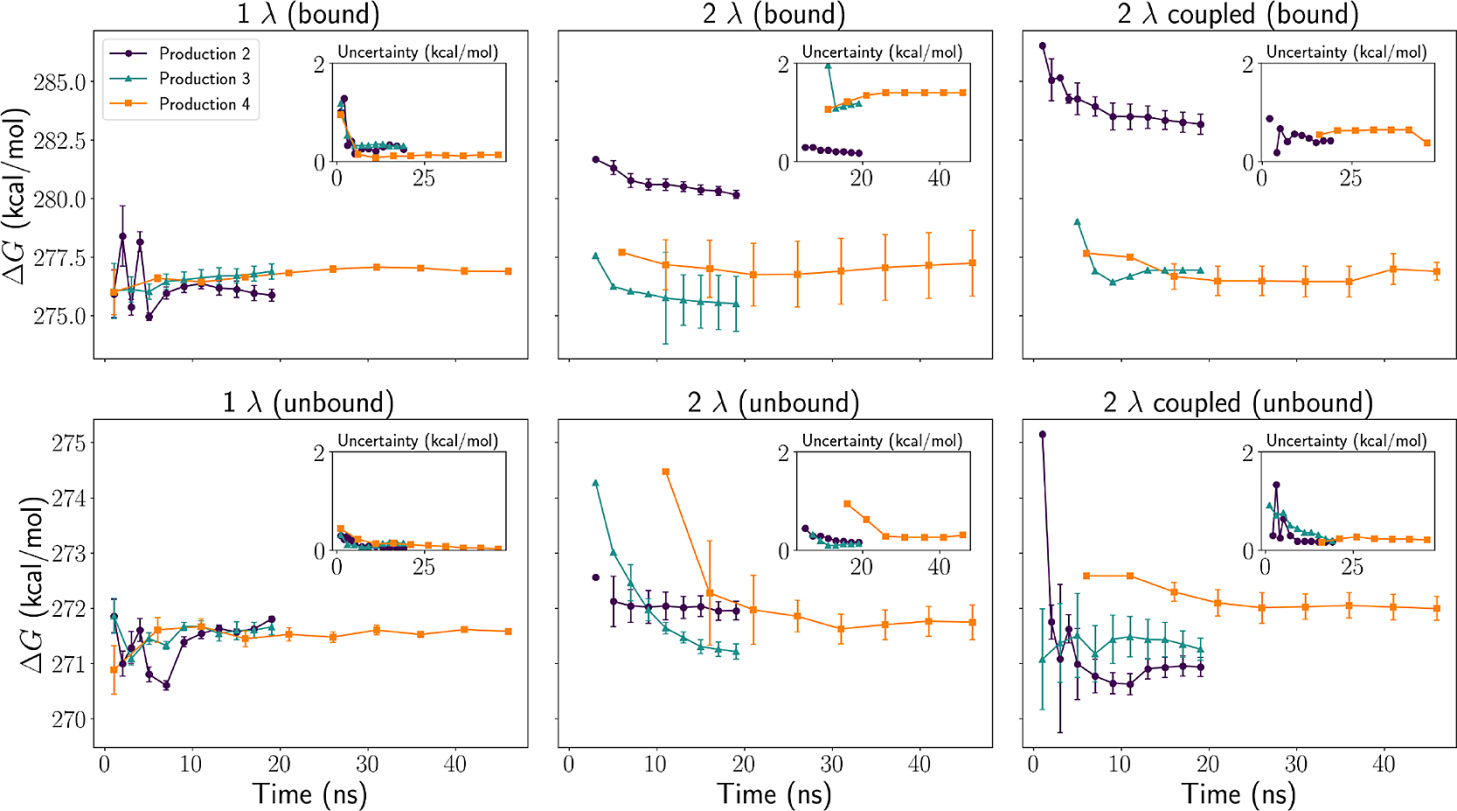
Free energy difference Δ*G* and corresponding uncertainties across all production runs for all perturbation schemes. The purple line corresponds to Production 2, the blue line to Production 3, and the orange line to Production 4. The insets show the magnitude of the uncertainty over the trajectory for all three runs.

The mutational free energies from native to nonspecific sequences in each ensemble and the corresponding uncertainties up to 50 ns of simulation time are shown in Figure 3. The full 100 ns trajectory for Production 4 is detailed in Supplementary Figure S4.

The 1λ scheme exhibited rapid stabilization of Δ*G* values, closely matching those observed in Production 4 (Δ*G* ≈ 277 kcal/mol for the bound system and Δ*G* ≈ 272 kcal/mol for the unbound system). Uncertainty values converged quickly, reaching approximately 0.2 kcal/mol for the bound system in Production 2 and 0.08 kcal/mol in Production 4. For the unbound system, uncertainty took longer to converge but ultimately stabilized at 0.03 kcal/mol in Production 4, indicating that the 1λ scheme enables efficient and precise Δ*G* convergence.

In contrast, the 2λ scheme required significantly longer sampling times, failing to reach convergence until Production 4. By then, Δ*G* values for the bound system had stabilized around 277 kcal/mol, similar to the 1λ scheme, albeit with slightly higher uncertainties (approximately 0.4 kcal/mol bound and 0.1 kcal/mol unbound). This suggests that while the 2λ scheme can achieve comparable precision to the 1λ approach, it does so at a higher computational cost. The coupled 2λ scheme exhibited even slower convergence, with uncertainty values remaining substantially higher in Production 4 (0.8 kcal/mol bound, 0.2 kcal/mol unbound). This indicates that coupling the λ values did not improve convergence but instead led to excessive exploration of sequence space, reducing sampling efficiency for the final matched states.

Despite differences in transition counts (Figure 2), both the 1λ and 2λ schemes achieved similar precision in Production 4. As discussed in the previous section, the 2λ scheme undergoes fewer transitions overall but maintains sufficient transitions to the final matched states, allowing free energy values to converge despite reduced sampling efficiency. In contrast, the coupled 2λ scheme, which distributes transitions across all sequence states, exhibits slower convergence and higher uncertainty due to undersampling of the states of interest.

While the 2λ scheme explores a broader conformational space than the 1λ scheme, its efficiency must be weighed against its computational cost. From Figures 3 and S4, we estimate that the 2λ scheme required approximately 80 ns into Production 4 to reach convergence, corresponding to a total of 1210 ns of simulation time (76 ns per sequence). In contrast, the 1λ scheme converged within the first 20 ns of Production 3, requiring only 460 ns in total (115 ns per sequence). If maximizing sequence space exploration is the primary objective, the 2λ scheme is preferable. However, in most practical applications, mismatched base mutations are not the primary focus. When excluding mismatched states, the effective cost of the 2λ scheme increases to approximately 300 ns per sequence—more than twice that of the 1λ scheme. Thus, while the 2λ scheme is advantageous for broader sequence exploration, its significantly higher computational cost makes the 1λ scheme the more practical choice for free energy calculations.

Overall, the 1λ scheme has proven to be the most efficient in terms of convergence speed and error precision for these Δ*G* calculations. The final binding free energy differences, , are summarized in Table 1. By Production 4, all schemes converged to an average ΔΔ*G*_*b*_ of approximately 5.7 ± 0.3 kcal/mol, with the 1λ scheme exhibiting the lowest uncertainty, followed by the 2λ scheme and, finally, the coupled 2λ scheme.

**Table 1:** Binding Free Energy Values ΔΔ*G*_*b*_ in kcal/mol for Each Production Run^a^

	production 1	production 2	production 3	production 4
1λ	4.1 ± 0.3	4.0 ± 0.3	5.6 ± 0.4	5.6 ± 0.2
2λ	10.8 ± – –	–– ± – −	4.7 ± 1.2	6.1 ± 0.4
coupled 2λ	–– ± – –	–– ± – −	5.3 ± – −	5.5 ± 0.8

a Values that could not be determined due to insufficient sampling are represented by – −.

The converged ΔΔ*G*_*b*_ values closely align with experimental results^65^ and are consistent with previous steered MD studies.^54^ Although the latter employed the AMBER14SB/BSC1 force fields while the present study used CHARMM36m, the discrepancies fall within 1 *k*_*B*_*T*, indicating that the observed differences are within thermal fluctuations. Notably, the previous study required approximately 4 μs of simulation time per sequence, whereas our approach achieved comparable accuracy with only 1.4 μs total (350 ns per sequence). Given that the 1λ scheme achieved sufficient transitions within about 100 ns of sampling per sequence, future work may explore an adaptive stopping criterion to optimize simulation efficiency.

### Estimating ΔΔ*G*_*b*_ for Other Mutants

Upon determining that the 1λ per base pair protocol outperforms the other methods tested, we applied it to investigate two additional mutations: from W-box (GGTCAA) to GGACAA and from W-box to GGTCCG. These mutations have been previously characterized experimentally, with ΔΔ*G*_*b*_ values reported in the literature.^65^ The final results are summarized in Table 2.

**Table 2:**
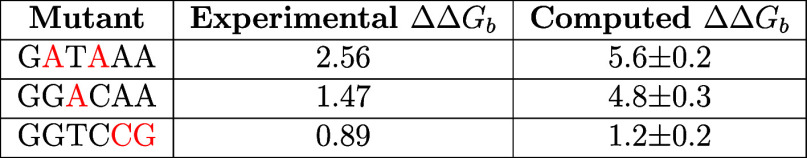
Comparison of Experimental and Computed ΔΔ*G*_*b*_ Values (kcal/mol)

The results show a successful ranking of the binding affinities of each mutant sequence. This was achieved despite the relatively small differences in experimental ΔΔ*G*_*b*_ values among the systems. Specifically, the smallest difference was 0.58 kcal/mol, observed between GGACAA and GGTCCG, while the largest was 1.67 kcal/mol, observed between GATAAA and GGTCCG. This level of precision indicates that the method could be highly valuable for screening binding sites and accurately predicting the effects of DNA mutations on the binding affinity.

It is important to note that the ΔΔ*G*_*b*_ values obtained were systematically larger than experimental values. One possible reason for this discrepancy could be technical sources of error, such as not sampling at equilibrium or underestimating the uncertainties in the measurements. Given that over 100 transitions occurred during Production 4 and that the ΔΔ*G*_*b*_ values converged within 10 ns, equilibrium sampling is unlikely to be the predominant source of error. On the other hand, it is known that bootstrapping underestimates uncertainties when data points are correlated.^66^ In typical MD simulations, some degrees of freedom can take microseconds to tens of microseconds to relax,^67^ which makes sampling statistically independent data points unfeasible. Nonetheless, the underestimation of the uncertainty is unlikely to be the main issue.

A more important factor could be the difficulty in fully capturing the biophysical changes that should occur after mutation. In previous studies^68,69^ on protein–DNA binding free energies where DNA mutations were introduced using alchemical methods, a good correlation between experimental and computed ΔΔ*G*_*b*_ values was demonstrated.^69^ Additionally, these studies found that ΔΔ*G*_*b*_ did not correlate with changes in the number of hydrogen bonds (HBs) or salt bridges, and that mutations did not significantly alter DNA base step parameters, suggesting that the full molecular mechanisms are not being completely captured. This potentially stems from either the force field parameters not allowing for changes^68,68^ in HBs or salt bridges or sampling limitations.

Both reasons would be challenging to address. In particular, for the latter, relevant conformational changes may not occur until microseconds or even milliseconds, a time scale not feasible in our simulations. For example, a 10-microsecond all-atom MD simulation of WRKY bound to W-box and nonspecific GATAAA sequences showed a reorientation of the protein after 4 μs, which led to lowering the binding free energy.^54,57^ This reorientation was attributed to a switch from a “recognition” (i.e., specific) mode of binding to a “search” (i.e., nonspecific) mode of binding. In our 100 ns production run, no such reorientation occurred, and we did not detect changes in the configuration of protein–DNA HB contacts (Supplementary Figure S5). However, despite the lack of large-scale conformational shifts, we observed a notable reduction in HB occupancy for mutations associated with larger ΔΔ*G*_*b*_ values: when mutating GGTCAA to GATAAA, average occupancy dropped from 38 to 26%, and for the mutation GGTCAA to GGACAA, it declined from 51 to 26%. These findings suggest that while the native sequence is sampled in its energetic ground state, the mutated sequence may remain trapped in a metastable conformation due to insufficient sampling time. If longer time scale dynamics were accessible, the system might further relax into a lower-energy state, potentially decreasing ΔΔ*G*_*b*_ and improving agreement with experimental values.

To assess whether λ-dynamics is sensitive to such conformational changes, we performed simulations on the rotated, nonspecifically bound structure obtained after 10 μs of MD. This resulted in a dramatic reduction of ΔΔ*G*_*b*_ to 0.4(0.2) kcal/mol, a value within thermal fluctuations. This confirms that the conformational change led to a loss of sequence specificity, flattening the free energy landscape. The discrepancy between our original and rotated-state simulations suggests that the native sequence preferentially stabilizes the specific binding mode, whereas the mutated sequence favors the nonspecific rotated state. The true ΔΔ*G*_*b*_ likely falls between these two computed values, but determining it accurately would require either extended simulations long enough to sample transitions between the two states or treating them separately and measuring ΔΔ*G*_*b*_ from the specific to the nonspecific rotated state.

Additionally, effects from sequences flanking the binding site may also impact the results. A recent study has shown that sequences flanking the binding site can affect the measurements of the dissociation constant *K*_*D*_ and, consequently, the relative binding free energy.^54^ Given that the experimental measurements were taken on a 15 base pair DNA construct and the core binding site is only 6 base pairs, flanking sequences are expected to significantly affect the measurements and could result in lower ΔΔ*G*_*b*_ estimates due to additional binding/unbinding events on the flanking sequences. Overall, these findings suggest that while our method is effective at ranking binding affinities and shows promise for drug screening, further refinements may be necessary to accurately explain experimental measurements of ΔΔ*G*_*b*_.

Another strength of λ-Dynamics lies in its ability to assess multiple mutations within a single simulation. For instance, in mutating GGTCAA to GATAAA, one can also obtain ΔΔ*G*_*b*_ for GATCAA and GGTAAA. Analogously, when mutating GGTCAA to GGTCCG, one can obtain ΔΔ*G*_*b*_ for GGTCAG and GGTCCA (Table 3). This allows identifying mutations that have the greatest impact on ΔΔ*G*_*b*_, thereby determining the dominant interactions within the protein–DNA complex.

**Table 3:**
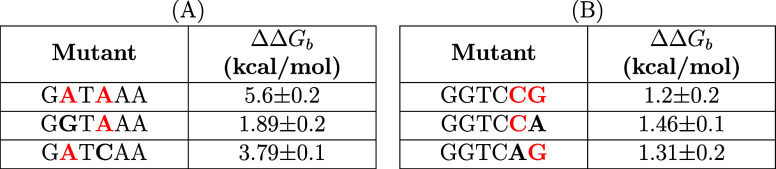
ΔΔ *G*_*b*_ for Each Mutation^a^

a The substituents are shown in bold, and the mutated bases are shown in red. (A) Shows the ΔΔ*G*_*b*_ values obtained from mutating GGTCAA → GATAAA. (B) shows those obtained from mutating GGTCAA → GGTCCG.

For instance, upon mutating from GGTCAA to GATAAA the ΔΔ*G*_*b*_ values suggest that the GATCAA (≈4 kcal/mol) exerts a more substantial destabilizing influence than GGTAAA (≈2 kcal/mol). In contrast, when we consider consecutive base changes, such as those from GGTCAA to GGTCCG, the relationship between individual mutations and the total binding affinity appears to be nonadditive. Each single-base mutation results in roughly the same ΔΔ*G*_*b*_ (1.3 kcal/mol for GGTCAG and 1.5 kcal/mol for GGTCCA), indicating that the overall destabilization of the system may be governed by similar biophysical processes irrespective of which base is mutated.

This study was limited to investigating three mutant sequences within the same simulation, primarily to evaluate the effectiveness of different λ variation schemes and to compare the method’s effectiveness in determining ΔΔ*G*_*b*_ values against previously used methods.^54^ However, the method is scalable, albeit with important caveats.

As the number of perturbation sites increases and the combinatorial space exceeds several hundred sequences, sampling challenges become more pronounced. Even in an ideal scenario, where the free energy landscape has been completely flattened and transitions are equally distributed among all *N*_*s*_ substituents (as observed in the 1λ scheme and the coupled 2λ scheme, see Supplementary Table S3), sampling requirements would scale linearly with *N*_*s*_ and exponentially with the number of sites. At the observed rate of about 1–2 transitions per ns, simulations would need to run for hundreds of nanoseconds to microseconds to achieve similar convergence for about 10 mutant sequences.

To address the scalability challenges, the Potts model estimator has recently been implemented.^70^ The Potts model simplifies free energy calculations by considering only one-body terms and two-body interactions (or couplings) between sites. This approximation reduces sampling requirements, allowing the Potts model to scale quadratically with the number of sites rather than exponentially. However, this comes at the cost of neglecting third and higher-order couplings between sites, which may limit its accuracy in more complex systems. In our case, where we mutated at most two sites, the histogram-based estimator is expected to perform similarly to the Potts model.^70^

It is worth mentioning that out-of-equilibrium alchemical simulations can also be an efficient and accurate tool for large-scale protein–DNA affinity measurements. In a previous study,^29^ 16 proteins were alchemically mutated, and the free energies were calculated from out-of-equilibrium transitions using the Crooks fluctuation theorem.^71^ The primary advantage of such an approach is the efficiency of the simulations: ΔΔ*G*_*b*_ values were obtained from approximately 40 ns of sampling time per sequence, compared to our estimated 200 ns of sampling per sequence. However, that study focused on mutations at a single site, whereas λ-Dynamics can explore multiple substituents at multiple sites within the same simulation, potentially allowing for greater efficiency. The study also reported an average correlation coefficient of 0.56, which did not improve with longer trajectories. In our case, the correlation with experimental data for the WRKY system was high, but further studies on different TFs are necessary to obtain a reliable estimate of the correlation with experimental data.

## Conclusions

In conclusion, this study demonstrates the efficacy of the 1λ per base pair protocol in Multisite λ-Dynamics for predicting protein–DNA binding affinities. The method demonstrated superior convergence, precision, and efficiency when compared to other perturbation schemes, successfully ranking binding affinities despite systematic overestimation of ΔΔ*G*_*b*_ values. While current discrepancies between computations and experiments may arise from sampling limitations, the scalability and accuracy of this approach make it highly promising for large-scale DNA binding studies. Future work will focus on extending the application of this method to a broader range of protein–DNA systems to further validate its predictive power.

## Supporting Information Available

Details on ALF potentials and flattening protocol; supplementary figures on transitions, free energy, and hydrogen bonding patterns; and example scripts are available for download at https://github.com/RyanLeeHayes/PublicationScripts/blob/main/2025TranscriptionFactors.tgz (PDF)
