# Supplementary material for: Multisite λ-Dynamics for Protein–DNA Binding Affinity Prediction
Source: J Chem Theory Comput. 2025 Mar 24;21(7):3536–44. doi: 10.1021/acs.jctc.4c01408 (PMC11983716; doi:10.1021/acs.jctc.4c01408)
Supplement: Supplementary file 1 — ct4c01408_si_001.pdf [file ct4c01408_si_001.pdf]

Supporting Information: Multisite λ-Dynamics for Protein-DNA Binding Affinity Prediction

Carmen Al Masri,[†] Jonah Z. Vilseck,[‡] Jin Yu,[*,¶] and Ryan L. Hayes[*,§]

†*Department of Physics and Astronomy, Uninversity of California, Irvine, California 92697, United States*

‡*Department of Biochemistry and Molecular Biology, Center for Computational Biology and Bioinformatics, Indiana University School of Medicine, Indianapolis, Indiana 46202, United States*

¶*Department of Physics and Astronomy, Department of Chemistry, University of California, Irvine, California 92697, United States*

§*Department of Chemical and Biomolecular Engineering, Department of Pharmaceutical Sciences University of California, Irvine, California 92697*

E-mail: jin.yu@uci.edu; rhayes1@uci.edu

S-I Forms of Biasing Potentials

The biasing potentials V_{Bias} follow the functional forms previously described in.[1,2] These potentials are scaled by coefficients ϕ, Ψ, ω, and χ, as detailed in Eqs. S-1, S-2, S-3, and S-4, respectively. The coefficients are tuned using the ALF method (described in Section S-II: Flattening Protocol) for each site s ($s = 1, \ldots, M$, where M is the total number of sites) and for every pair of substituents i and j ($i, j = 1, \ldots, N_s$, where N_s is the number of substituents at site s), as follows:

- The fixed bias, ensuring that the end points have similar free energies and can be sampled within the same simulation:

$$V_{Fixed} = \sum_{s}^{M} \sum_{i}^{N_s} \phi_{si} \lambda_{si} \tag{S-1}$$

- The quadratic potential, flattening the roughly parabolic barrier found in regions between $0.1 \leq \lambda \leq 0.9$:

$$V_{Quad} = \sum_{s}^{M} \sum_{i}^{N_s} \sum_{j>i}^{N_s} \Psi_{si,sj} \lambda_{si} \lambda_{sj} \tag{S-2}$$

  This term represents the interaction between substituents on the same site s, while V_{Quad} in Eq.(5) corresponds to an interaction term between substituents on separate sites s and t.

- The end point potential, ensuring the flattening of the deep free energy wells at $\lambda = 0$ and $\lambda = 1$ due to solvent cavity getting disrupted:

$$V_{End} = \sum_{s}^{M} \sum_{i}^{N_s} \sum_{j>i}^{N_s} \omega_{si,sj} \frac{\lambda_{si} \lambda_{sj}}{\alpha + \lambda_{si}} \tag{S-3}$$

  Where $\alpha = 0.017$ was found to give good fits to the free energy for a broad range of substituents.

- The skew potential, which improves fits to free energy profiles obtained with soft-core interactions:

$$V_{Skew} = \sum_{s}^{M} \sum_{i}^{N_s} \sum_{j>i}^{N_s} \chi_{si,sj} \lambda_{sj} (1 - \exp(-\lambda_{si}/\sigma)) \tag{S-4}$$

where $\sigma = 0.18$

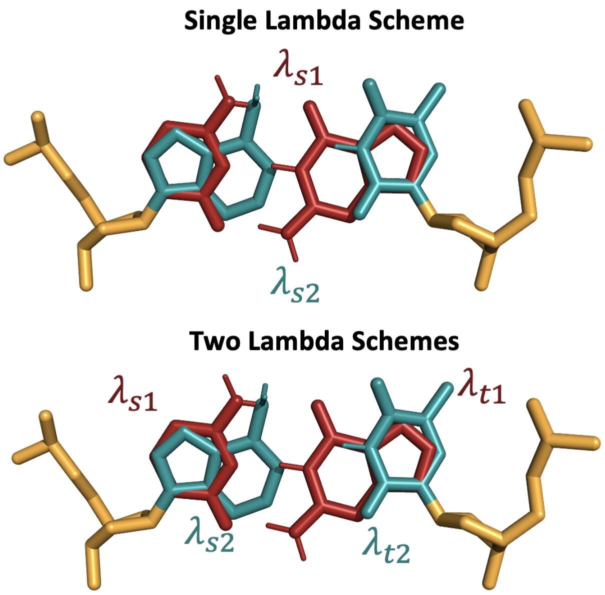

Figure S1: Setup of substituents at a given DNA site. The native substituent is shown in red, and the mutant substituent in blue. The sugar-phosphate backbone, representing environment atoms, is shown in yellow. In the single λ scheme (top), each base pair is scaled by a single λ: λ_{s1} for the native group and λ_{s2} for the mutant group. In the two-λ schemes (bottom), each base is scaled independently: at site s, the native and mutant groups are scaled by λ_{s1} and λ_{s2}, respectively, while at site t, they are scaled by λ_{t1} and λ_{t2}. .

S-II Flattening Protocol

The flattening of the free energy landscape closely follows that in a previous work[2] and is summarized below:

1. Free energy profiles are computed from multiple recent sampling iterations using the Weighted Histogram Analysis Method (WHAM)[3]

2. The entropy of the implicit constraints on λ is subtracted[4]

3. A scoring function is used to compute the change in biasing potential parameters using a least squares approach.[1] The scoring function is given by:

$$E = \sum_{p}^{profiles} \sum_{b}^{bins} \frac{w_{pb}}{2} (G_{pb} + \Delta G_{pb} - \bar{G}_p)^2 + \sum_{i}^{biases} \frac{k}{2\gamma_0^2} \Delta\gamma_i^2 \tag{S-5}$$

$$\Delta G_{pb} = \sum_{i}^{biases} \frac{\partial G_{pb}}{\partial \gamma_i} \tag{S-6}$$

Where $\gamma_i \in \{\phi_i, \Psi_{si,sj}, \chi_{si,sj}, \omega_{si,sj}\}$ are the biases, $k = 1(\text{kcal/mol})^2$ and $\gamma_0 = \{2, 8, 2, 1\}$ kcal/mol the regularization parameters and w_{pb} the weight associated with the corresponding bin.

The initial term in Equation S-5 evaluates the flatness of the landscape and predicts how changing the biasing parameters will impact this flatness. The second term prevents overfitting during consecutive flattening runs. The equation is minimized using least squares, with $\bar{G}_{pb}$ and $\Delta\gamma_i$ as free parameters.

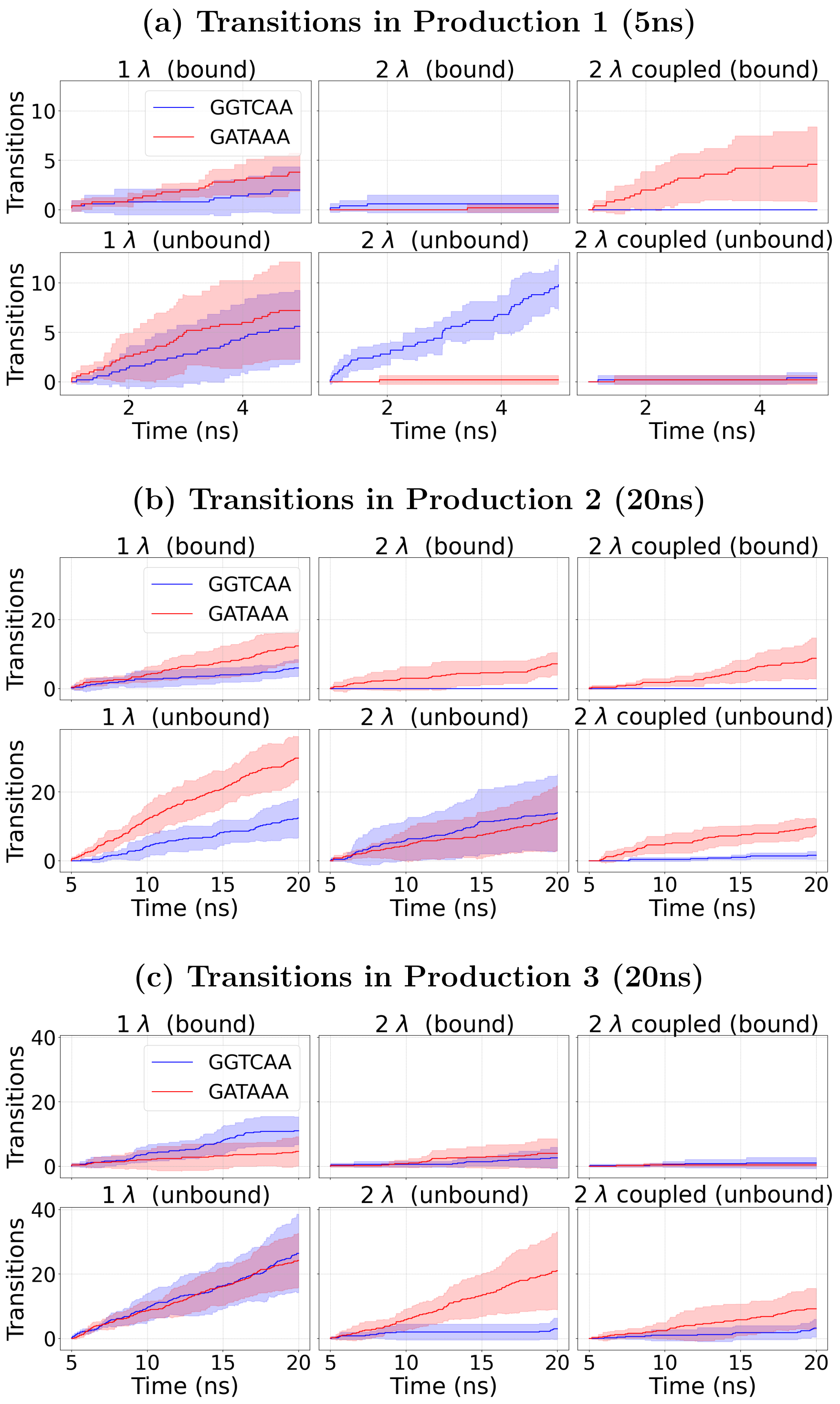

Figure S2: The average number of transitions to the specific DNA sequence GGTCCG (blue) and the nonspecific GGACAA sequence (red) is shown for all three perturbation schemes during (a) Production 1, (b) Production 2, and (c) Production 3. The shaded region represents the standard deviation across 5 runs. Results for the bound system are shown in the top row, while those for the unbound DNA are shown in the bottom row.

S-III Total Transitions

Bound

Strand 1<br>Strand 2	GGTCAA<br>CCAGTT	GGTAAA<br>CCATTT	GATCAA<br>CTAGTT	GATAAA<br>CTATTT	GGTAAA<br>CCAGTT	GGTCAA<br>CCATTT	GATCAA<br>CCAGTT	GGTCAA<br>CTAGTT	GATAAA<br>CCAGTT	GGTCAA<br>CTATTT	GATCAA<br>CCATTT	GGTAAA<br>CTAGTT	GATCAA<br>CTATTT	GGTAAA<br>CTATTT	GATAAA<br>CTAGTT	GATAAA<br>CCATTT	Total
**1 λ/ bp**	52(28)	48(19)	63(12)	57(18)	N/A	N/A	N/A	N/A	N/A	N/A	N/A	N/A	N/A	N/A	N/A	N/A	**220(40)**
**2 λ/bp**	31(9)	29(21)	16(8)	6(5)	0(0)	3(2)	15(6)	0(0)	0(0)	0(0)	1(0)	0(0)	1(0)	0(0)	0(0)	21(18)	**122(31)**
**2 λ/bp + coupling**	6(4)	5(6)	2(0)	5(3)	8(12)	47(41)	4(4)	2(0)	3(2)	15(11)	38(37)	19(18)	33(16)	12(10)	11(8)	4(3)	**214(64)**

Unbound

Strand 1<br>Strand 2	GGTCAA<br>CCAGTT	GGTAAA<br>CCATTT	GATCAA<br>CTAGTT	GATAAA<br>CTATTT	GGTAAA<br>CCAGTT	GGTCAA<br>CCATTT	GATCAA<br>CCAGTT	GGTCAA<br>CTAGTT	GATAAA<br>CCAGTT	GGTCAA<br>CTATTT	GATCAA<br>CCATTT	GGTAAA<br>CTAGTT	GATCAA<br>CTATTT	GGTAAA<br>CTATTT	GATAAA<br>CTAGTT	GATAAA<br>CCATTT	Total
**1 λ/ bp**	113(17)	164(10)	117(14)	160(9)	N/A	N/A	N/A	N/A	N/A	N/A	N/A	N/A	N/A	N/A	N/A	N/A	**554(26)**
**2 λ/bp**	131(15)	13(10)	132(14)	13(10)	0(0)	5(2)	1(0)	7(3)	0(0)	1(0)	1(0)	(0)	4(2)	1(0)	0(0)	1(0)	**309(26)**
**2 λ/bp + coupling**	10(5)	12(10)	13(5)	10(8)	16(10)	34(42)	5(3)	11(4)	9(7)	9(8)	36(39)	34(20)	30(32)	18(14)	22(11)	8(6)	**277(74)**

Figure S3: Total number of transitions to each physical state in Production 4 for the bound (top) and unbound (bottom) systems when mutating GGTCAA to GATAAA.

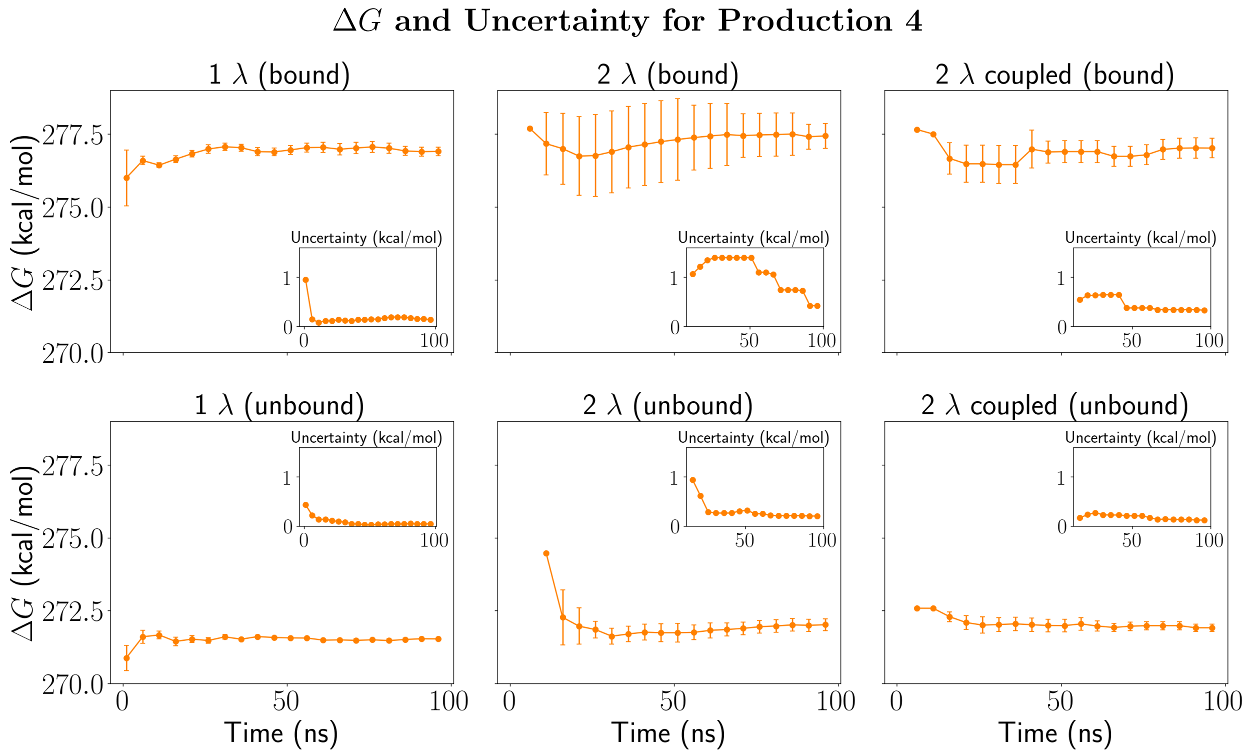

Figure S4: Free energy difference ΔG and corresponding uncertainties for Production 4 for all perturbation schemes. The insets show the magnitude of the uncertainty over the trajectory.

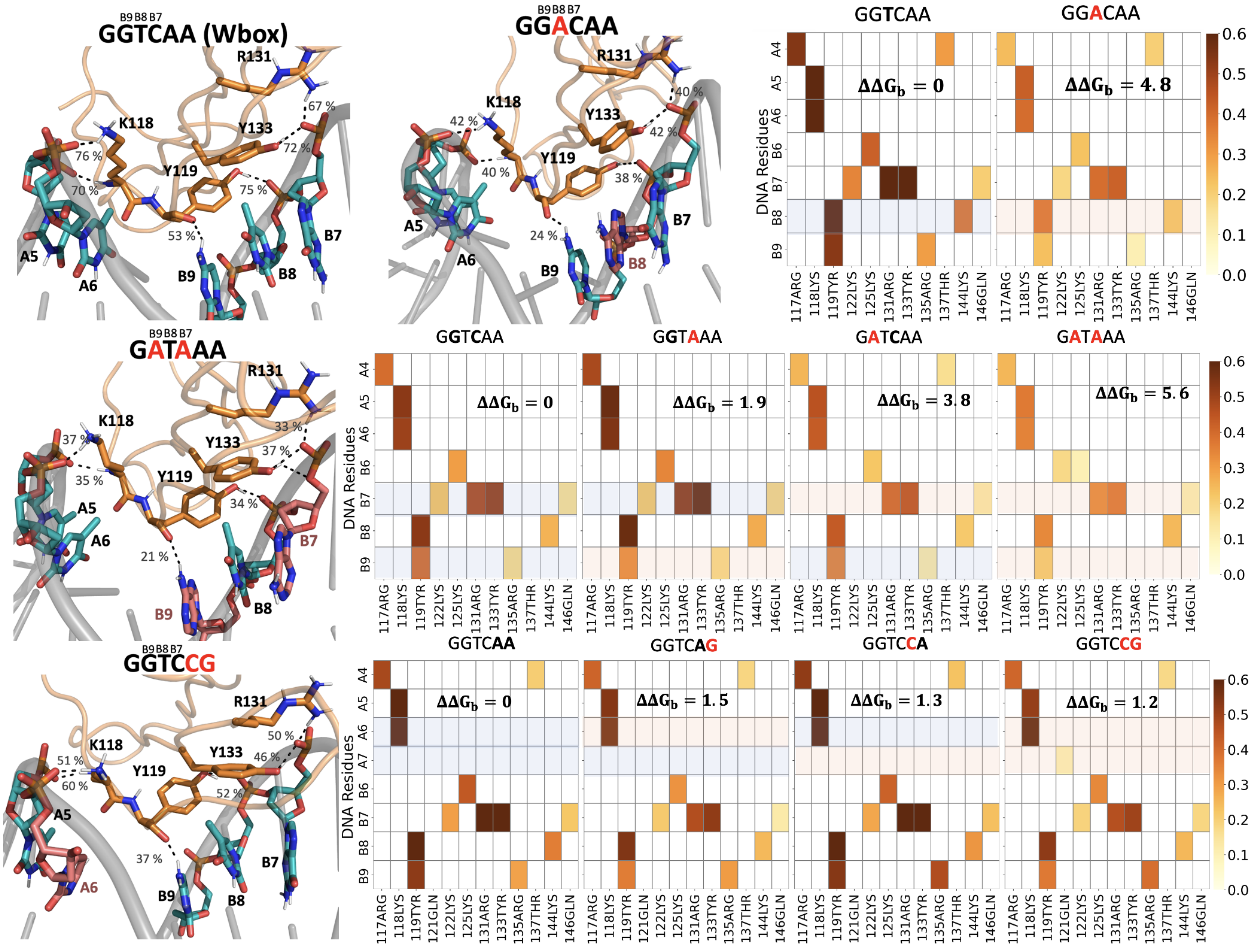

Figure S5: Heatmaps of protein-DNA HB occupancies for all mutants, with molecular visualizations of key protein-DNA interactions. In the heatmaps (right), the x-axis represents protein residues, and the y-axis shows DNA residues. Rows corresponding to the mutation sites are highlighted in blue for the native substituent and red for the mutant substituent. The $\Delta\Delta G_b$ values for each mutation are displayed on the corresponding heatmap. The DNA sequences are indicated above each heatmap, with mutations highlighted in red. The molecular visualizations (left) show key HB interactions, with gray percentages indicating the occupancy of each HB contact.

References

(1) Hayes, R.L., Armacost, K.A., Vilseck, J.Z., and Brooks III, C.L., 2017. Adaptive landscape flattening accelerates sampling of alchemical space in multisite λ dynamics. *The Journal of Physical Chemistry B*, 121(15), pp.3626-3635.

(2) Hayes, R.L., Vilseck, J.Z., and Brooks III, C.L., 2018. Approaching protein design with multisite λ dynamics: Accurate and scalable mutational folding free energies in T4 lysozyme. *Protein Science*, 27(11), pp.1910-1922.

(3) Kumar, S., Rosenberg, J.M., Bouzida, D., Swendsen, R.H. and Kollman, P.A., 1992. The weighted histogram analysis method for free-energy calculations on biomolecules. I. The method. *Journal of computational chemistry*, 13(8), pp.1011-1021.

(4) Knight, J.L. and Brooks III, C.L., 2011. Applying efficient implicit nongeometric constraints in alchemical free energy simulations. *Journal of computational chemistry*, 32(16), pp.3423-3432.
